# The Anatomical and Histological Dissection of the Human Ear in the Normal and Diseased Condition

**Published:** 1892-06

**Authors:** 


*The Anatomical and Histological Dissection of the Human Ear in the Normal and Diseased Condition.* By DR. ADAM POLITZER. Translated by GEORGE STONE. Pp. xv., 269. London: BAILLIÈRE, TINDALL AND COX. 1892.

Everything that comes from the pen of Dr. Politzer is worthy of perusal, for he is, indeed, a master of his subject; but the book before us is probably the best thing he has ever written, and seems to leave very little to be said on the anatomy of the ear. It gives evidence of the most laborious and painstaking work, and is characteristic of the Teutonic mind. Since the days of Toynbee, very little original work on this subject has been done by Englishmen; and it is to Germans that we look for all recent investigations in normal and pathological anatomy of the ear. We are, therefore, under special obligations to Mr. George Stone of Liverpool for translating this book into such excellent English. The illustrations are nearly all from original dissections made by the author, and are, many of them, of a beautiful character—not the tinted diagrammatic drawings which so often do duty in text-books of diseases of the ear, but accurate copies of dissections, which may be relied upon to give a true idea of the actual parts. The book will take its place as the best standard work on the subject.

